# Multi-Sensor Orientation Tracking for a Façade-Cleaning Robot

**DOI:** 10.3390/s20051483

**Published:** 2020-03-08

**Authors:** Manuel Vega-Heredia, Ilyas Muhammad, Sriharsha Ghanta, Vengadesh Ayyalusami, Siti Aisyah, Mohan Rajesh Elara

**Affiliations:** 1 Engineering Product Development, Singapore University of Technology and Design, 8 Somapah Road, Singapore 487372, Singapore; milyasmeo@gmail.com (I.M.); ghanta1996@gmail.com (S.G.); vengadeshavio91@gmail.com (V.A.); siti2_aisyah@sutd.edu.sg (S.A.); rajeshelara@sutd.edu.sg (M.R.E.); 2 Department of Electrical Engineering, UET Lahore, NWL Campus, Lahore 54890, Pakistan

**Keywords:** reconfigurable robots, glass-façade-cleaning robots, multi-sensor integration, robot heading tracking, orientation tracking

## Abstract

Glass-façade-cleaning robots are an emerging class of service robots. This kind of cleaning robot is designed to operate on vertical surfaces, for which tracking the position and orientation becomes more challenging. In this article, we have presented a glass-façade-cleaning robot, *Mantis v2*, who can shift from one window panel to another like any other in the market. Due to the complexity of the panel shifting, we proposed and evaluated different methods for estimating its orientation using different kinds of sensors working together on the Robot Operating System (ROS). For this application, we used an onboard Inertial Measurement Unit (IMU), wheel encoders, a beacon-based system, Time-of-Flight (ToF) range sensors, and an external vision sensor (camera) for angular position estimation of the *Mantis v2* robot. The external camera is used to monitor the robot’s operation and to track the coordinates of two colored markers attached along the longitudinal axis of the robot to estimate its orientation angle. ToF lidar sensors are attached on both sides of the robot to detect the window frame. ToF sensors are used for calculating the distance to the window frame; differences between beam readings are used to calculate the orientation angle of the robot. Differential drive wheel encoder data are used to estimate the robot’s heading angle on a 2D façade surface. An integrated heading angle estimation is also provided by using simple fusion techniques, i.e., a complementary filter (CF) and 1D Kalman filter (KF) utilizing the IMU sensor’s raw data. The heading angle information provided by different sensory systems is then evaluated in static and dynamic tests against an off-the-shelf attitude and heading reference system (AHRS). It is observed that ToF sensors work effectively from 0 to 30 degrees, beacons have a delay up to five seconds, and the odometry error increases according to the navigation distance due to slippage and/or sliding on the glass. Among all tested orientation sensors and methods, the vision sensor scheme proved to be better, with an orientation angle error of less than 0.8 degrees for this application. The experimental results demonstrate the efficacy of our proposed techniques in this orientation tracking, which has never applied in this specific application of cleaning robots.

## 1. Introduction

Cleaning and maintenance of modern buildings is one of the most important tasks of service robots. In the last decade, different technologies have been developed and tested to achieve this goal. Robots that climb on vertical surfaces have been developed for such applications. Commonly, robots are used for buildings, bridges [1], pipes [2,3], and power lines [4]. In the same way, they are used for maintenance applications: Wall maintenance [5], power line maintenance [6], and wind tower maintenance [7]. These robots are tele-operated. The technology development for glass-façade cleaning in urban building environments is growing drastically; e.g., self-cleaning glass [8] and sol-gel coating [9].

The vertical-surface-climbing robots have various locomotion types, which include, for instance, track wheels, multiple-legged frames [10], and sliding frames [11]. They also include various adhesion techniques [12], such as grippers, blowers, passive cups [13], and active negative suction pressure cups Tun et al. [14] and Nazim et al. [15]. The commercial robots for window-cleaning applications are growing fast, such as Winbot X, Winbot 950, Winbot 850, Hobot, and Alfawise, etc. However, none of these robots can transition from one window panel to the other due their design limitations. These platforms need sensory systems to have better performance, and the building infrastructure also needs to be modified by installing some kind of sensor network for deployment of robots in cleaning tasks [16].

One of the fundamental aspects of any mobile robotic system is the determination of the correct orientation for stabilizing itself and reaching the goal position. To obtain the orientation angle, there are some existing methods, including the use of a camera for visual orientation tracking [17,18], omni-directional imaging [19], accelerometers and gyros [20], fish-eye lenses [21], digital compasses [22], distance sensors [23], pointed targets [24], segmented maps [25], and other methods [26]. There are some existing robots that can function without any orientation sensors as well [27]. These robots can be branched out into various applications based on the basic information given by the platform, i.e., its orientation. For localization, there are also efforts using visual techniques [28,29] and estimation techniques [30,31]. After obtaining the orientation angle data, the next step is to control the orientation of the robot to ensure the robustness and resilience of the overall system [32,33,34,35].

The focus of this paper is the orientation estimation of glass-façade-cleaning robots that can be equipped with suction and passive sweeping systems. The orientation of cleaning robots is an important topic because the suction and sweeping systems have a specific direction of operation. If the robot is not aligned correctly, then the cleaning will not be adequate and efficient. In addition, in most robots, given the mechanism of locomotion, the orientation plays an important role in controlling the direction of the robot’s movement, critical during transitions between window panels.

The proposed glass-façade-cleaning robot (*Mantis v2*) [36] is designed to climb vertical surfaces; this robot uses an active suction mechanism and is used for window cleaning and glass inspection [37]. It is vital to maintain the orientation of *Mantis* robot during navigation on window frame, panel transitions and during lifting and displacement of modules. The most critical system of *Mantis* is the attachment control using the vacuum pump [38]. The suction is affected by the surface’s variation and robot’s pad collisions. The energy is linked to the time it takes the robot to cover the entire surface of the glass window. In addition, if the orientation of the robot is unknown, it would cause the robot to be more susceptible to unwanted movements and collisions with the window frame, which could lead to a fall backwards and possible damage of the robot and the working environment. Moreover, the *Mantis* robot has the ability to make the transition from one window panel to the other. During the transition phase, it is critical to maintain the robot alignment to avoid any collision with the obstacle (e.g., the window frame).

This article describes in detail the mechanical and electrical design of *Mantis v2*, as well as the installation of different sensory systems for orientation angle estimation and, hence, the achieved stabilized operation during locomotion on vertical surfaces.

To develop a robust orientation system for a robot which can make transitions from one window panel to another while maintaining a desired angular position, five different sensory systems were evaluated for orientation tracking in this work:

- Encoders (locomotion systems)
- Time-of-Flight sensors (ToF)
- Sonar beacons
- Vision systems (cameras)
- Inertial measurement units (IMUs)

Section 2 describes the robotics platform and the sensory system of *Mantis*. The position and orientation control are described using this mechanism to correct and maintain the orientation. The applications of different sensors to estimate orientation of the *Mantis* and their limitations are described in the following Section 3. System integration using the Robot Operating System (ROS) is presented in Section 4. Section 5 gives details on the experimental setup and a discussion of the results, followed by the conclusions of this work in Section 6.

## 2. Hardware Description of the Façade-Cleaning Robot: *Mantis*

### 2.1. Overview of the Mantis v2 Robot

**Figure 1:**
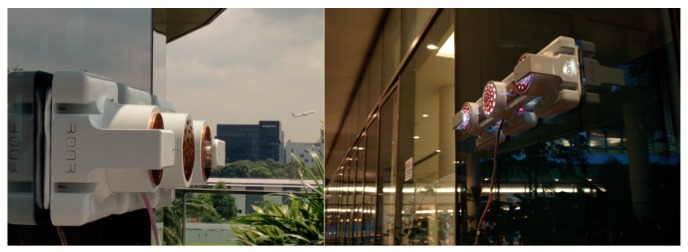
A situation in which the *Mantis* cleans a glass window panel of a building.

*Mantis v2* is a robot consisting of three modules that are interconnected through longitudinal bars of carbon-fiber material, which maintains each of the modules in its respective position. The modules are separated from one another by a distance of 30 cm, allowing it to cross over positive obstacles like window frames. A real picture of the *Mantis* robot working on a glass window is shown in Figure 1.

**Figure 2:**
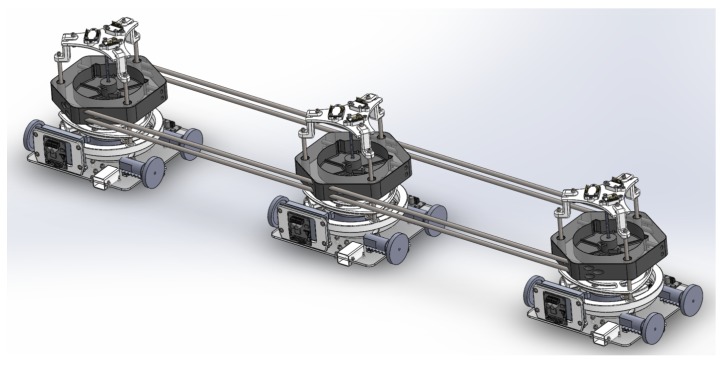
Modular design of the *Mantis V2* robot.

Each module has a rotational mechanism, enabling the robot to freely rotate about its central axis. This rotational movement is limited to $90^{\circ }$ counterclockwise. When the pad is attached to the surface of the window, it is able to rotate independently from the longitudinal bar because of the slip-ring (see Figure 2). This rotation is caused by the locomotion mechanism, which is described in Section 2.3.

The base of the robot is made using a 3 mm acrylic sheet and 3D printed parts using polylactic acid (PLA), thermoplastic polyurethane-elastomere (TPU), and carbon fiber. The principal differences between *Mantis v1* [36] and *Mantis v2* are the types of sensors, the materials used to build them, and their locomotion mechanisms.

The prototype uses an external power supply, as it requires 24 volt continuously, which is used to power up the blower. In addition, a voltage regulator connected to the main power source supplies 12 volts for the sensing, control, and locomotion systems.

**Figure 3:**
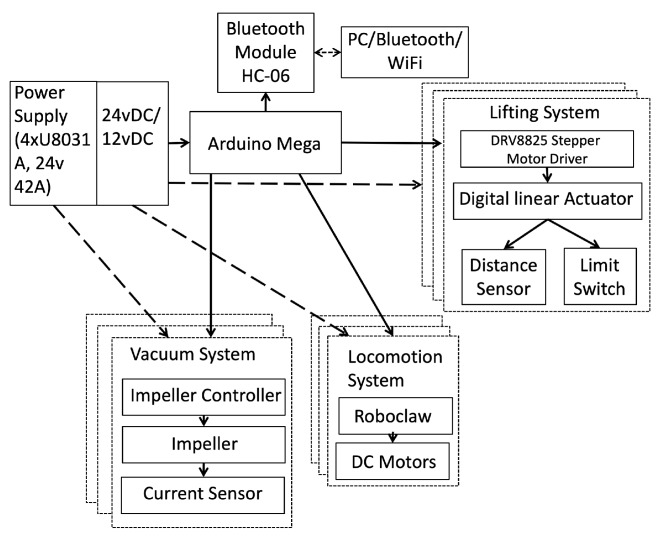
Diagram of the system architecture for *Mantis*’s control.

The architecture diagram (Figure 3) describes the *Mantis*’s electronic module. The entire functioning of this robot is controlled by a 16 bit micro-controller unit (MCU), Arduino Atmega 2560, which is responsible for controlling the robot functions and establishing a wireless communication protocol between the robot and operator by serially using an HC-06 Bluetooth module as well. It receives commands from the operator and sends control signals to the blower controller, stepper motor driver, and the Roboclaw speed driver controlling the DC motors based on the received commands.

The DC motors communicate through serial port communication. Multiple Roboclaw speed controllers can be connected to a single node of a full-duplex communication. Every instruction packet that is transmitted contains the respective motor ID, which helps to control each motor individually on a single node of communication. The stepper motor driver (DRV8825) is used to drive the linear actuator, which is responsible for lifting the mechanism. The distance sensor helps to estimate the height to which the module is lifted, and the limit switch helps to cut down the lifting function when it reaches the maximum position. The robot performs the cleaning by means of a microfiber towel placed on the bottom of the pad in contact with the glass (e.g., [36]). The details are not explained in this paper. In the following subsections, the major modules of the *Mantis v2* robot are described briefly.

### 2.2. Locomotion Mechanism

The locomotion mechanism of the *Mantis v2* robot consists of DC motors with 360 degrees of continuous rotation, a stall torque of 1.5 Nm, and a no-load speed of 60 rpm. Each actuator and wheel is located equidistantly from the center of the adhesion cup, hence balancing the normal forces equally between all four wheels. The design of the locomotion mechanism of the robot makes it nearly holonomic.

### 2.3. Rotational Mechanism

The system can rotate itself using a rotating ring mounted in the center of the module, causing it to be secured firmly between the tapered roller bearing mechanisms, while being able to rotate about the center of the module. This is shown above in Section 2.1. This allows the ring to act as a connecting point between the module and the robot structure. The ring has three carbon fiber bars, positioned equidistantly from each other. The rotation of each module is limited to $90^{\circ }$ clockwise and counterclockwise to simplify the control of the robot. The angular positions of these modules are sensed by $360^{\circ }$ rotational encoders. At the same time, IMU sensors are placed in each module to measure the absolute orientation of the module relative to the body of the robot. The module’s rotation is also achieved using the wheels positioned in each module, explained in Section 3.1.

### 2.4. Transition Mechanism

As a cleaning robot, one of *Mantis*’s unique skills is its ability to make a transition from one window panel to another, crossing over the frame between the panels. This eliminates the need for manual transfer of the cleaning robots from one panel to the other, as is done with conventional window-cleaning robots.

In order to achieve this, a transverse re-positioning mechanism was developed using linear actuators. The actuators separate a module from the surface on which *Mantis v2* resides, moving each module one by one across the obstacle (window frame). This sequential movement of modules on the façade prevents the robot from losing its grip on the surface and falling down.

### 2.5. Suction System

The main holding force of the *Mantis v2* robot comes from the suction pads. Given the area of the suction pad, the blower generates a vacuum to ensure that the robot does not fall or slide on the surface of the glass window. The impeller inside the blower generates a maximum vacuum pressure of 8 kPa. The operational voltage of the blower motor speed controller is from 16 to 27 V. The rotational velocity of the blower motor can be controlled using a potentiometer or from the MCU. In order to ensure that the suction loss is minimized, the suction pad is made of PLA, and a rubber skirt is pasted around the edges to seal the vacuum area.

### 2.6. Mantis’s Sensors

The sensory system of the *Mantis v2* is used to sense and control the orientation of the robot during navigation and the transition of the robot from one panel to the other. In addition, there are three distance sensors on each module. One of the distance sensors is used to measure the height of the module base from the glass surface during the lifting process. The other two sensors, directed towards the surrounding structure of the window, are used to determine the orientation of the robot using the shape of the window, as explained in Section 3.1.

**Figure 4:**
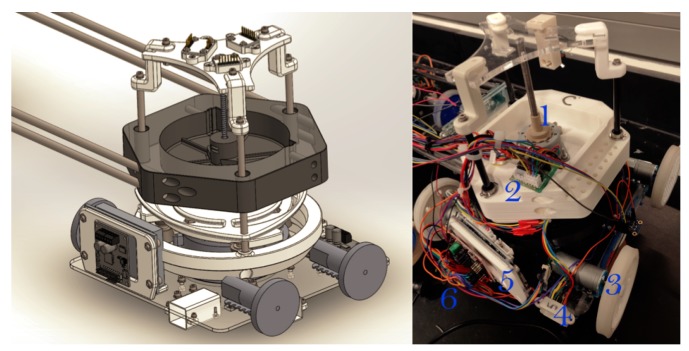
*Mantis*’s module C. 1. Linear actuator, 2. speed driver for the linear actuator, 3. locomotive mechanism, 4. distance sensor, 5. speed controller of the blower, 6. speed controller of the locomotive actuators, and 7. inertial measurement unit (IMU).

The placement of the blower, suction cup, and actuators mounted on the acrylic platform is shown in Figure 4. Each module consists of multi-sensory systems, e.g., distance sensors, IMUs, encoders, etc. The distance sensors are positioned on the upper and lower sides of each module and placed at a position lower than the height of the frame.

In the same way, the each module has an IMU to measure the orientation of the individual modules Figure 4. To know the orientation of the main axis of the robot, another IMU sensor is placed on the top of the robot. This helps to estimate the orientation of each module and the overall absolute orientation of the main axis of the robot body. The operation is explained in Section 3.5. Other sensors used to estimate orientation of *Mantis* are sonar beacons. Two receiver beacons are placed on top of the first and third modules in order to get position coordinates, which are used to calculate orientation as per Equation (10). An external monitoring camera is fixed on a supportive rig.

## 3. Orientation Estimation

The most common form of window panels is square or rectangular. Regardless of the shape in the upper part, the bottom is always flat and parallel to the floor. Given the locomotive characteristics and the navigational path of the *Mantis v2* in zig-zag, the orientation of the robot should always be maintained parallel to the floor.

However, at the time of making the transition between the window panels, it loses surface contact, and it is observed that, due to the weight of the module, the robot tends to change its orientation. In the same way, during the displacement on the surface of the glass, the caterpillar actuators lose traction due to dirt or humidity on the window, which means that the displacement is not continuous and linear, which causes disorientation of the robot as well. To maintain the stability of the *Mantis v2*, this change in orientation must be estimated and corrected.

The importance of maintaining the orientation of the robot during the transition is due to the fact that in some window frames, the width exceeds 12 cm; this is the transition limit. The robot consists of a sensory system to identify and avoid hitting the frame [36]. If the robot moves in a non-planar way during the transition, it runs the risk of hitting the frame, losing the suction, and falling down as a consequence.

### 3.1. Locomotive Orientation

**Figure 5:**
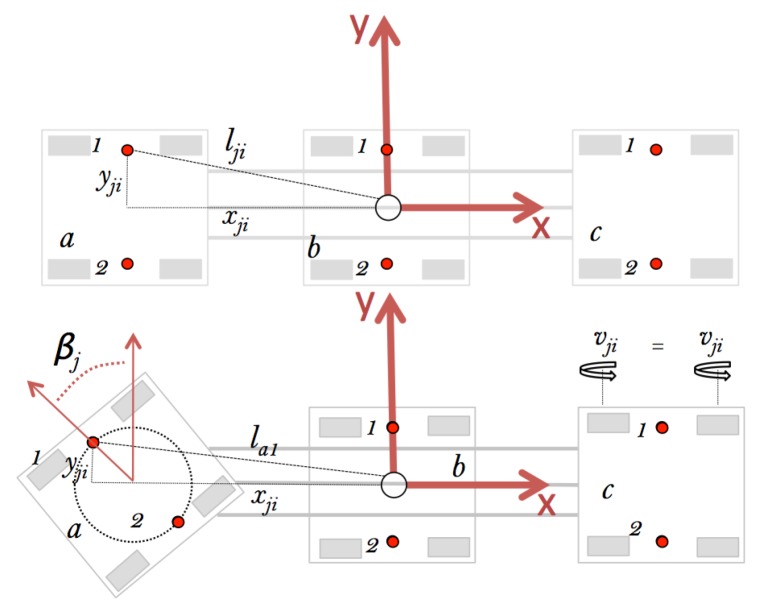
Diagram of the *Mantis*’s actuators; the red circle shows the middle point between the actuators of each side *i* (*i* = 1,2) and the position from the inertial system of the robot positioned in the central module.

*Mantis v2* is an omnidirectional robot on flat surfaces. Each of the pads can freely rotate $360^{\circ }$ using the wheels controlled by the DC motors. Currently, the movement is limited to $90^{\circ }$. When the pads rotate, the position of the actuators changes relative to the center of rotation of the module, affecting the robot’s locomotion. Therefore, for the position of the actuators $l_{ji}$ (Equation (1)) in relation to the centroid of the *Mantis*’s body, the fixed distance between the modules $d_{j}={\left(x,y\right)}^{T}$ and the distance *e* from the centroid of the module j are considered, where j=a,b,c and the actuators’ sides i=1,2, as shown in Figure 5.

(1) $$l_{ji}=\left(\begin{array}{c} x_{ji}\\ y_{ji} \end{array}\right)=\left(\begin{array}{c} d_{a_{x}}+e\cos\left(\beta_{j}\right)\\ d_{a_{y}}+e\sin\left(\beta_{j}\right) \end{array}\right)$$

The orientation of the actuators is directly related to the angle $\beta_{j}$, which gives the orientation to the pad and therefore to actuators 1 and 2 and to each pad asynchronously. The linear velocity *V* in Equation (2) of the robot is calculated as:

(2) $$V=\frac{1}{6}\sum_{j=a,i=1}^{j=c,i=2}v_{ji}.$$

To know $v_{ji}$, Equation (3) is used as follows:

(3) $$v_{ji}=\varphi_{ji}*2r=\frac{\left({\left(t-1\right)}_{m}-t_{m}\right)}{2(1x10^{6})}*GR*2r,$$

where $\varphi_{ji}$ is the angular velocity of the actuator, ${\left(t-1\right)}_{m}-t_{m}$ is the time for the encoder pulse in μs, GR is the motor/gear box relation, and *r* is the radius of the wheel. So, the combined interaction of the actuators gives the linear velocity to the robot. In the calculation of the angular velocity ω, the following Equation (4) is used, given the position $l_{ji}$ of the actuator and the direction of the displacement speed $\alpha_{ji}$:

(4) $$\omega =\frac{\left({\hat{v}}_{ji}\cos\left(\beta_{j}\right)\cos\left(\sum_{j=a,i=1}^{j=c,i=2}\alpha_{ji}\right)\right)}{\sum_{j=a,i=1}^{j=c,i=2}l_{ji}},$$

where ${\hat{v}}_{ji}$ is the differential velocity between modules 1 and 2. Equations (3) and (4) are used for the control of the robot, simulated in Section 5.

#### Problems with the Encoder’s Readings during Displacement

Given the conditions on the windows, during the navigation of the robot on the window, specifically when it moves towards the top of the window, a skidding phenomenon is frequently observed during the locomotion. Since the wheel encoder’s readings are used to calculate the position of the robot during locomotion, it exhibits erroneous behavior in orientation and position calculation due to the skidding of the *Mantis v2* on the window panel.

During the operation of the blower, small vibrations are generated in the whole structure of the robot. These vibrations produce erroneous readings on the sensors as well. To alleviate this problem, rubber cushions are placed to absorb these vibrations and reduce false readings. However, occasionally, these vibrations cause false measurements from the on-board sensors, especially from the wheel encoders.

### 3.2. Orientation Using Time-of-Flight (ToF) Sensors

**Figure 6:**
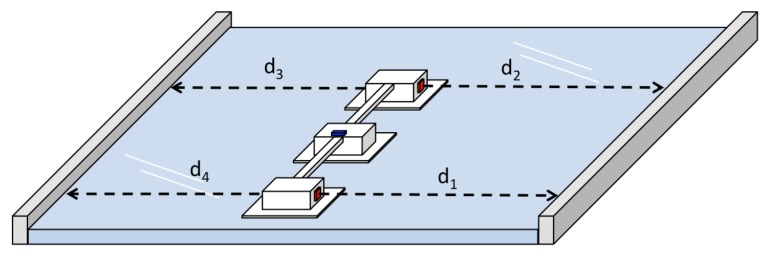
Sensor positions: The red mark refers to the distance sensor placement and the blue mark refers to IMU.

One of the methods used to estimate the orientation of the *Mantis v2* in this work is to use the ToF sensor’s range data with respect to the window frame on both sides of the robot. This method is based on the distance of the lateral modules of the robot to the lower and upper parts of the frame of the window, as shown in Figure 6.

**Figure 7:**
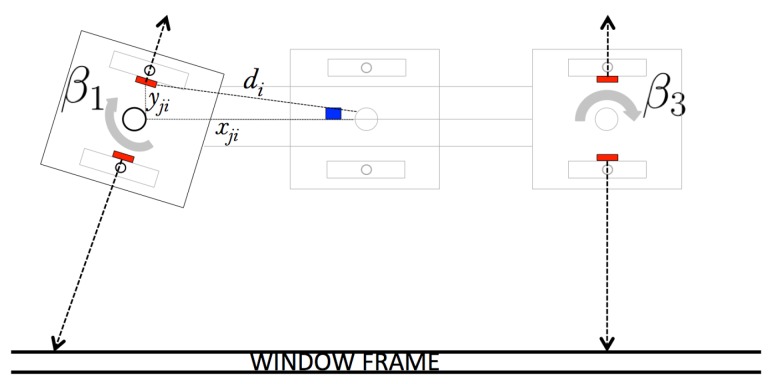
Rotation of the pad $\beta_{1}$ in relation to the rest of the modules, $\beta_{2}$ and $\beta_{3}$; this modifies the alignment of the pads and, therefore, the measurement point.

However, each of the pads can rotate about its own axis in the center of the pad, modifying the orientation of the pad in relation to the body of the robot. Since each of the pads contains an IMU sensor, we can separately calculate the orientation $\beta_{j}$ of each pad, as shown in Figure 7. Additionally, each pad has a joint encoder to estimate the angular position of the pad. The rotating capacity of each of the pads is mechanically limited to $90^{\circ }$.

To determine the distance to the window frame, the ToF sensor’s beam readings are used. Given the distances between the modules and each orientation $\beta_{j}$, it is possible to calculate the slope of the robot relative to the lower and/or upper frame of the window.

(5) $$d_{i}=\cos\beta_{j}ds+\sin\beta_{j}ds$$

**Figure 8:**
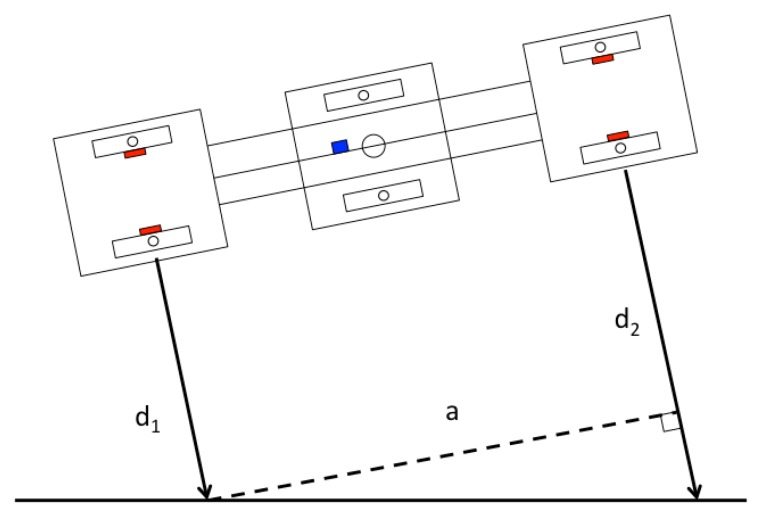
Straight alignment of the modules to estimate the orientation angle, which can be corrected by knowing the value of $\beta_{j}$.

For the measurement obtained from each of the ToF sensors $d_{i}$ (s=1,2,3,4), as given in Equation (5), the correction is applied based on the orientation $\beta_{j}$ of the individual pad. In this way, it is possible to estimate the distance of the pads to the frame to make alignment corrections, as illustrated in Figure 8.

To increase the orientation accuracy, two angles are calculated, $\theta_{1}$ and $\theta_{2}$, one with respect to the upper frame and the other to the lower frame. Then, by taking the average of $\theta_{1}$ and $\theta_{2}$ in Equations (6) and (7), we can reduce the high-frequency noise caused due to vibrations during locomotion of the *Mantis v2* robot.

(6) $$\theta_{1}=\arctan\left(\frac{d_{2}-d_{1}}{a}\right),$$

and, in the same way, for

(7) $$\theta_{2}=\arctan\left(\frac{d_{3}-d_{4}}{a}\right),$$

given $\theta_{1}$ and $\theta_{2}$,

(8) $$\psi_{ToF}=\frac{\sum \theta_{i}}{i},$$

where *a* is the distance between the sensor positions $d_{1}$ and $d_{2}$ (i.e., the length of the robot).

Given the orientation angle of the robot $\psi_{TOF}$, in Equation (5), it is corrected to a reference angle (i.e., $0^{\circ }$, parallel to the window frame), modifying the angular speed ω of the *Mantis* from Equation (4) when varying the speed of the locomotive actuators.

Equation (4) controls the angular velocity of the robot, considering the kinematic constraints of the robotic platform. The differential speed of the actuators in each module ${\hat{v}}_{ji}$ modifies the steering angle $\beta_{j}$ of each pad and, therefore, the direction of the displacement of the whole module by adjusting the orientation of the *Mantis v2*.

#### Problems with ToF

The ToF distance sensors detect the frame boundary from a height of 1.5 cm. The sensor placement height can be adjustable to 1 cm, constrained to the robot construction. Due to safety concerns, the ToF sensors were placed at a height of 1.5 cm. Sometimes the windows have no frame, or it is less than the height of the sensor placement; in some modern buildings, the frames of the windows are completely flat, or even with a negative slope. In these situations, the sensor does not work properly, since it can not detect the window frame. Given these inherent limitations, the ToF sensors occasionally produce false negative readings, which cause the robot not to detect the frame. If the current value is considerably different from the previous values, this is not taken into account, according to Equation (9).

(9) $$\mathrm{if}\;(d_{i_{t}}>2\left(\sum_{k=t-3}^{k=t-1}d_{i_{k}}\right)||0.5\left(d_{i_{t}}<\sum_{k=t-3}^{k=t-1}d_{i_{k}}\right))$$

$$d_{i_{t}}=d_{i_{t-1}}$$

### 3.3. Orientation Based on Sonar Beacons

**Figure 9:**
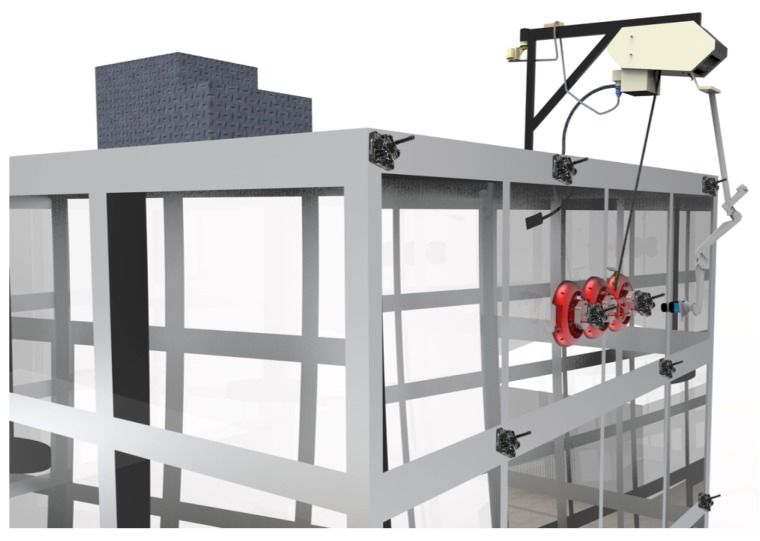
Beacon navigation system terminals on the glass façade structure of a building, in strategic corners of the windows.

There are recent trends towards the integration of technology into buildings. Modern buildings contain sensors of various types, such as relative humidity sensors, temperature sensors, barometric pressure sensors, etc. In the structures of glass façades, it is proposed to place a series of sensors strategically so that they help to navigate the robot on the entire façade see Figure 9.

**Figure 10:**
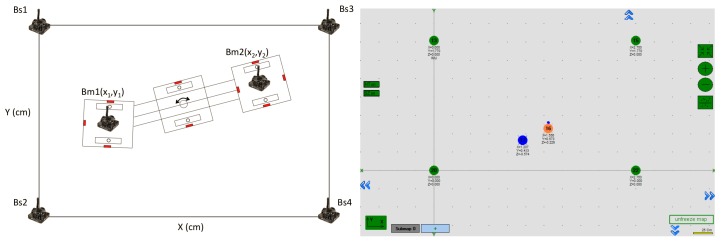
Beacon positions: The Bss are the static beacons and the Bms are the mobile beacons mounted on the *Mantis v2* in the left panel; in the right panel, the position of the beacons is shown in the MarvelMinds software: Static in green, and mobile in blue and orange.

The sensors used in this work were ultrasonic (sonar) beacons, which have a recommended intercommunication distance of up to 30 m. For the accomplishment of *Mantis*’s orientation tests, these beacons were placed at every three meters in the working environment. These sensors contain ultrasonic emitters and receivers in five directions. For each direction, the ultrasonic waves are sent and received within $90^{\circ }$ from their centers, as shown in Figure 10. Stationary beacons were arranged in such a way that the entire area was covered. Each beacon can be differentiated by a unique device ID. These beacons can be programmed as mobile or static devices in the software. When all of the beacons are arranged in known stationary positions, the receiver beacon can be placed on the robot, which operates inside the stationary beacons’ perimeter.

The cartesian coordinates of the mobile beacons (Bms) were estimated with help of the stationary beacons (Bss) via radio ranging. The communication between the beacons and the modem is established with radio frequency using a proprietary protocol with a 915 MHz frequency. The modem was programmed with the positions of the Bss. Based on the Bss’ known position values and the ranging data received, the modem estimated the position of the Bm with the help of a distance matrix, as shown in Table 1. The modem was connected to the laptop via USB–Serial communication. The software of the MarvelMind beacons helped to program the modem with the positions of the Bss.

The position for the Bs can be introduced to the software manually or can be taken automatically using the software from the MarvelMind. For this application, we captured the position of the Bs manually. The operation setup used in this study is shown in Figure 10.

Once the positions are programmed into the modem, it calculates the coordinates of the Bm at a defined update rate. With the help of predefined libraries from the ROS, the *Mantis v2* algorithm receives the cartesian coordinates of the Bms from the modem to estimate the orientation of the robot. The proposed algorithm to calculate the orientation of the robot on the glass façade using ranging measurement of two Bms fixed on external pads separated with a distance of 80 cm is given in Equation (10):

(10) $$\Psi_{B}=\arctan\left(\frac{Bm_{Y2}-Bm_{Y1}}{Bm_{X2}-Bm_{X1}}\right).$$

The orientation calculation process repeats each time that the ROS receives new data. The frequency of the main data is given by the beacon’s frequency and has a low data update in the ROS. The variable in the ROS for the position of the mobile beacons maintains the same value until it receives a new update.

#### Problems with the Beacon-Based Orientation Method

The battery life of the beacons mainly depends on the speed of the radio profile, which also affects the live coordinate update rate for the mobile beacon. Eventually, one or more beacons can lose connection with the main system. During this period, the orientation cannot be updated. It is necessary to charge the batteries in short periods during frequent use. The other issue observed in beacon-based positioning is the signal latency and intermittent obstruction during operation of these beacons.

### 3.4. Vision-Based Orientation

The façade-cleaning robots are assumed to operate in a controlled environment where we can set up sensors/systems beforehand to ease the navigation and monitoring of the *Mantis v2* robot. In this application, an external camera was installed to remotely monitor the working of the robot on a glass window, as depicted in Figure 9. We have made elegant use of this monitoring camera as an ’orientation tracking’ sensor as well. To calculate the orientation of the *Mantis v2* robot, the outer pads (modules) were covered with two red-colored circular plates. These circular covers were already part of the *Mantis* design to protect and cover the inner stuff of the robot.

**Figure 11:**
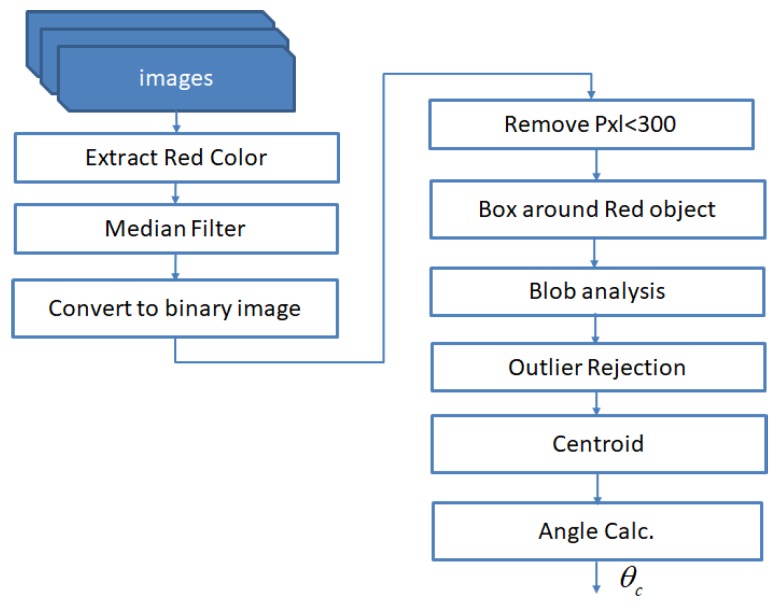
A flowchart to calculate vision-based orientation.

In the above algorithm (Figure 11), the red color filter is applied to extract the red-colored circular plate on the outer modules of the *Mantis v2* robot in the incoming image stream. To reject other red objects in the scene, a constraint of area and distance between the two plates is imposed to enhance the circular plate detection to ensure that only the red circular cover plates are detected in the image. Next, a median filter is applied to remove noise from the image. After this step, the pixels above than some threshold value are removed from the binary image. This step will leave only the red object in the image. The blob analysis technique labels the connected components and creates blobs of detected objects in the image. The most important step in orientation calculation lies in the extraction of centroid points in the detected red blobs in pixel coordinates. Here, as only two red cover plates are present in the processed image, their centroids are represented as $P_{1}\left(x_{1},y_{1}\right),P_{2}\left(x_{2},y_{2}\right)$.

The orientation calculation with respect to the camera’s horizontal axis is given by Equation (11). Note that the camera has already been aligned to the robot’s longitudinal axis during the calibration process, as described in Section 5.

(11) $$\psi_{c}=\arctan\left(\frac{y_{2}-y_{1}}{x_{2}-x_{1}}\right)$$

#### Problems with the Vision-Based Orientation Tracking

The external camera used to track the robot’s body needs to be aligned with the robot and should be free of any obstruction during the robot’s operation. These stringent requirements make the vision-based system a practically difficult option for robot orientation tracking. Similarly, operating in outdoor environments may cause light illumination issues and the casting of shadows by buildings.

### 3.5. Orientation Based on IMU

With the emergence of Micro-Electro-Mechanical Systems (MEMS) technology, the cost of inertial measurement unit sensors has been drastically reduced in the last decades. These sensors are being used in consumer-grade electronics product available in everyday applications. For example, IMUs are responsible for keeping track of the orientation and automatic screen tilting in mobile phones. An IMU consists of a triad of accelerometers and gyroscopes (also known as gyros) fixed at the orthogonal axis, with six degrees of freedom (DOF). Accelerometers sense accelerations and gyros sense rotation rates. However, these raw measurements need to be combined in an elegant way to get the desired information, e.g., to get the orientation out of IMU measurements. The measurements of this sensor are combined (fused) in such a way that we can benefit from its best features. Two very common approaches to fusing IMU data for orientation tracking are: Complementary filters (CF) and Kalman filters (KF) [39].

In this work, our goal is to estimate the orientation (angular position) of a façade-cleaning robot moving on a vertical surface (e.g., a glass window). As the operational environment is 2D, we can use angular rates about one axis only, i.e., measurements of an x-axis gyro. The angular change can be tracked by integrating the angular rate over the sampling time. To obtain the angular position with the accelerometer measurements, the gravitational acceleration sensed by the accelerometer is considered and, using simple trigonometric relationship, the tilt angle (heading angle in the case of the *Mantis* robot) is calculated. In this case, the outputs of the y-axis and z-axis accelerometers are used to calculate the tilt angle see Appendix A.1.

#### 3.5.1. The Problem with Accelerometers

As an accelerometer measures all of the forces that are acting on the platform on which an IMU is rigidly mounted, it will also sense a lot more than just the gravitational acceleration. Every small force working on the platform will disturb the accelerometer’s measurements. In the case of an actuated system (like *Mantis v2*), the forces that drive the system will be visible in the sensor output. The accelerometer data are reliable only in the long term, so a “low-pass” (LP) filter has to be used. However, using an LP filter will introduce latency into the calculated angle.

#### 3.5.2. The Problem with Gyroscopes

Gyroscope output can be used to obtain an accurate measurement of the angular position and is not susceptible to external forces. However, because of the mathematical integration process over time, the calculated orientation angle has the tendency to drift over time, i.e., not returning to zero when the system goes back to its original zero position. The gyroscope data are reliable only in the short term, as they start drifting with the long-term use.

#### 3.5.3. Sensor Fusion

**Figure 12:**
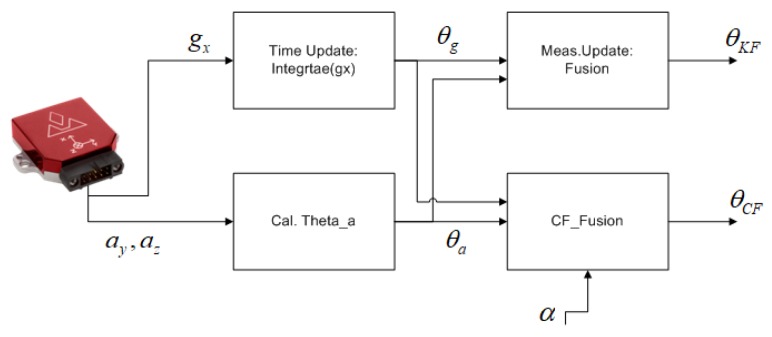
Complementary filter (CF) and Kalman filter (KF) processing for orientation estimation of the *Mantis v2* robot.

Sensor fusion is the process for mathematically integrating measurements of multiple sensors to achieve the best performance, as compared to individual sensors. Due to the limited computing resources on-board the robot (*Mantis v2*), the simplest readily available sensor fusion algorithms, i.e., the complementary filter (CF) and the one-dimensional Kalman filter (KF) see Appendix A.1, are used in this work. Mathematical formulations of these simple multi-sensor fusion algorithms are given in Appendix A, and a block diagram for the implementation of CF and KF in this paper is shown in Figure 12.

## 4. System Integration Using the Robot Operating System (ROS)

**Figure 13:**
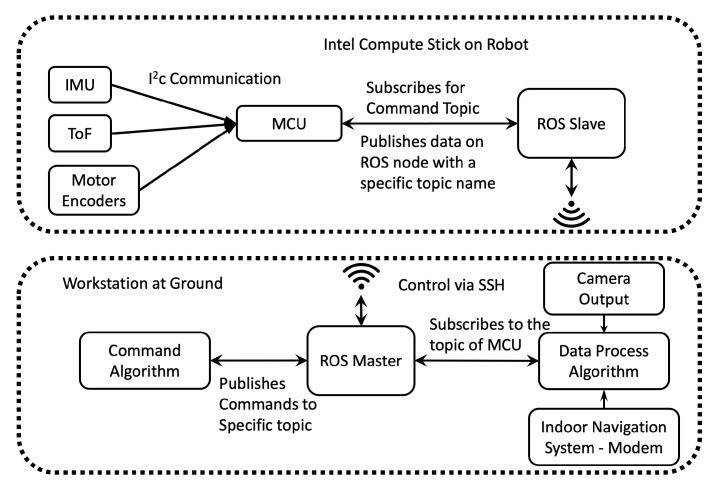
Robot Operating System (ROS)-*Mantis* sensor integration system.

In this work, the ROS is used to integrate different sensing systems from different brands. The ROS is a flexible framework for robot software integration. It is a collection of tools, libraries, and conventions that aims to simplify the task of creating complex and robust robot behavior across a wide variety of robotic platforms [40]. We used the Intel Compute Stick, which is a mini-computer, to host the ROS on-board and to transmit and receive the sensor data from *Mantis v2*. In the ROS, it is possible to create Master and Slave units, where the ROS on-board the robot acts as a Slave and the ROS at the base station acts as a Master. To create Master and Slave units, both systems should be connected to the same network. The robot’s terminal is controlled from the base system using Secure Shell (SSH) protocol. Accessing the terminal of the robot gives permission to control the ROS. Since the Master is at the base station, all of the required computation is carried out at the base station, while the robot publishes the acquired sensor data directly on an ROS node. The MCU on the robot is connected to the Intel Compute Stick with a USB cable. The ROS uses an existing serial node algorithm to establish communication between the MCU and ROS. The MCU is programmed to publish the sensor data on an ROS node with a specific topic name. On the other hand, a C++ algorithm is executed in the Master unit, which subscribes to the specific topic published by the MCU. Subscribing to the topic of the MCU means that the algorithm can obtain the sensor data and perform the required computations. The communication architecture is shown in Figure 13.

Using the different data obtained from the sensory systems, the integration of the sensors is made as follows in Equation (12):

$$\mathrm{if}\;(\psi_{Loc}>1.2\left(\frac{\psi_{TOF}+\psi_{B}+\psi_{IMU}+\psi_{C}}{4}\right)$$

(12) $$∥\psi_{Loc}<0.8\left(\frac{\psi_{ToF}+\psi_{B}+\psi_{IMU}+\psi_{C}}{4}\right))$$

(13) $$\theta_{R}=\frac{\psi_{Loc}+\psi_{ToF}+\psi_{B}+\psi_{IMU}+\psi_{C}}{5}.$$

The results of $\theta_{R}$ (Equation (13)) and the orientation data from different sensory systems are shown in Section 5.3.

## 5. Experimental Setup and Result Discussion

The experiments in this work were conducted using two methods: A test bed static experiment on a glass window frame, and a locomotion test during the robot’s navigation on the glass window.

**Figure 14:**
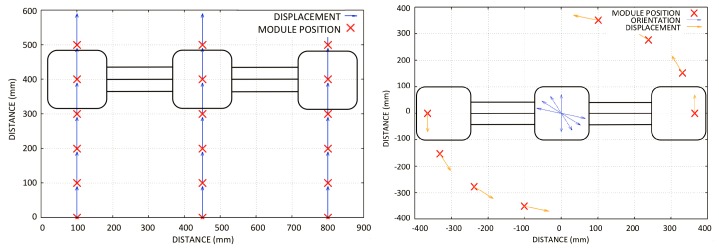
*Mantis*’s locomotion simulation: In the left panel, climbing the window; in the right panel, rotation of the robot; the red x shows the position of each module.

For the robot’s locomotion, the control developed using the Equations (3) and (4) is simulated in Figure 14.

### 5.1. Test Bed Experiments

**Figure 15:**
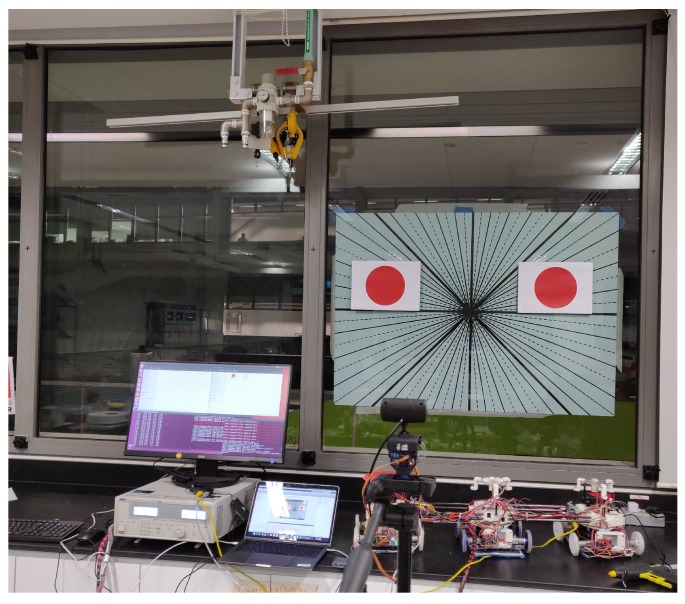
Test bed layout for sensor calibration in static positions.

To develop and verify algorithms, a test bed was set up, as shown below in Figure 15. As the operational environment is a 2D vertical surface in this work, the IMU was placed with the z-axis pointing up. This way, the output orientation angle is actually the roll angle, i.e., the angular rate about the x-axis was measured to obtain the heading (orientation) angle of the *Mantis v2* robot. The reason for this setup of the IMU is that, if we place this sensor vertically (x-axis pointing up/down), the heading angle (for comparison of results) of the *VN-100* IMU obtained from the z-axis is not reliable. The reason is that this heading angle is actually calculated with magnetometer data inside the IMU. The magnetometer measurements may be affected by metallic objects present around the working environment, e.g., metallic window frames. However, the roll angle is purely calculated from accelerometer and gyro data fusion inside the *VN-100* IMU, which is not affected by metallic objects around it.

Two red-colored circular paper sheets were pasted onto the test bed to be tracked with an external camera. Two Time-of-Flight (ToF) sensors were fitted on top of the test bed, so that laser rays emitted by the ToF sensors could strike the upper frame of the window. The distances measured by the two ToF sensors were used to calculate the orientation angle with respect to the horizontal frame of the window. Four sonar beacons were fixed around the test bed to get sound pings on the receiver beacon placed on the two outer modules of the robot on the test bed.

An angular graduated slate was placed on the back of the window glass, leveling it by using an analog leveler. The lines were separated every 10 degrees by placing intermediate dotted lines and increasing the thickness of the lines for 0, 45, 90 degrees. The camera was aligned with the horizontal line corresponding to 0 degrees on the slate. Using this slate, the offsets of the sensors, such as the beacons, camera, ToF sensors, and IMU, are corrected.

### 5.2. Static Tests

**Figure 16:**
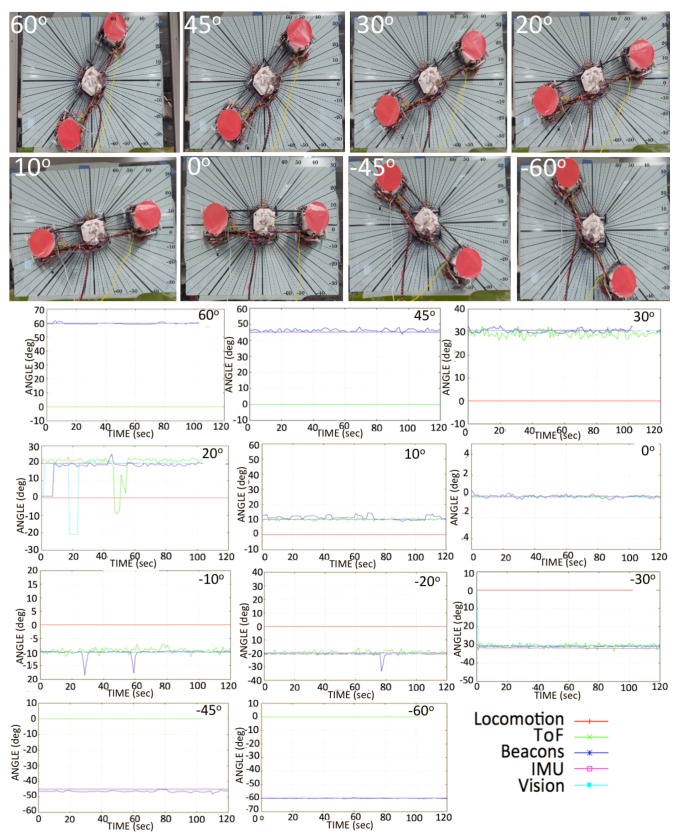
Experimental set-up for static orientations from $-60^{\circ }$ to $60^{\circ }$. (**Top**) *Mantis v2* robot (top view); (**Bottom**) Orientation sensor data plot for each static position.

After a previous calibration setup of the sensors, we captured multi-sensor data to verify the angular position accuracy of each sensory system, as shown in Figure 16. The test was conducted by placing the robot in static positions on the angular-graduated slate. The experiments ran for about 120 s at each angular position. In some angular positions, variations can be seen in sensor data, such as from the ToF sensors and beacons. Sensors such as the beacons tend to lose signal reception for three to five seconds, and ToF sensors occasionally sense false negative values from the window frame, which leads to an erroneous angular reading. A comparison of multi-sensory orientations with reference IMU data in static tests is shown in Figure 16 (bottom), and the numerical values of the angle errors are given in Table 2.

### 5.3. Dynamic Tests

In this test, the robot was moved on the façade with the tele-operated system. The robot was moved forward and rotated clockwise and counterclockwise in order to test the heading angle with different sensory systems.

**Figure 17:**
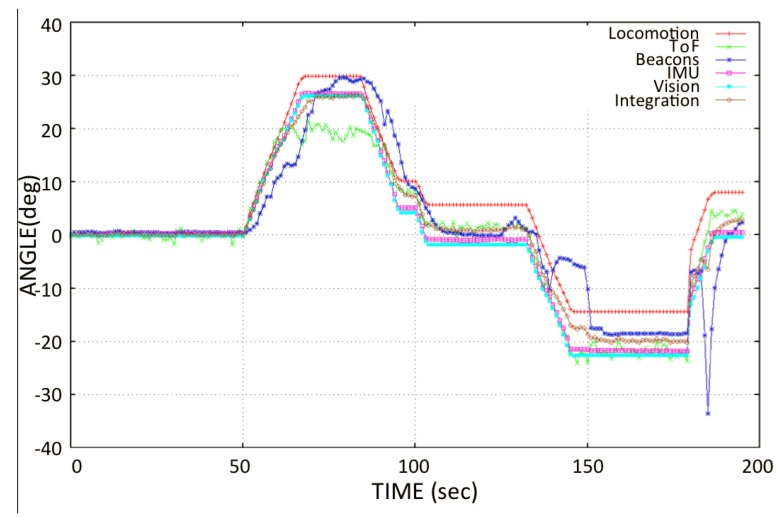
Navigation test: Orientation from different sensory systems during the robot’s angular movement.

In Figure 17, the behavior of all of the sensory systems is observed during the locomotion of the robot. The red line shows the movement of locomotion of the robot obtained through odometry. It was observed that over 50–65 s (approximately), the angle read from the locomotion had an offset, as compared with the real angular movement of the robot obtained from reference IMU. Subsequently, the skidding of the wheels on the glass increases the error between locomotion and the rest of the sensors. For instance, the locomotion system (wheel encoders) recorded an angular movement of about $10^{\circ }$ at the end of the experiment, whereas the true angular movement of the robot’s body was about $0^{\circ }$ at that instant. There was a delay in the data received from beacons; these have less stability, as compared to the IMU and vision sensors. In the same way, in the interval of about 60–90 s, an offset of ≈10${}^{\circ }$ in the the ToF sensor orientation was observed.

**Figure 18:**
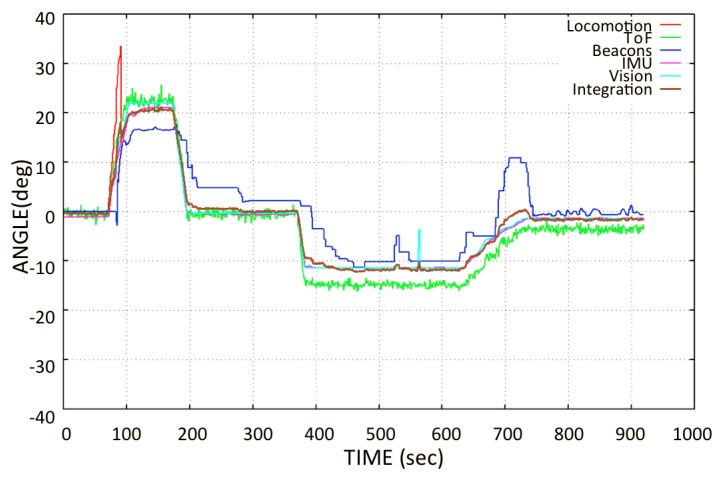
Transition phase test: Behavior of the orientation estimation during the transition phase when the pads turn off sequentially, shifting from one window panel to another.

In the same way, in Figure 18, a substantial delay was observed in the angle data from the beacons. In an actual hardware system, the incoming data have a frequency of 1 Hz, which means one datum per second. This data latency problem comes from the beacon system. The integrated angle looks good because the sensor integration suppresses the high-frequency noise from different sensors by averaging the orientation data, as given in Equation (19). In this plot, it is observed that the locomotion values are higher than those of the rest of the sensors. This is because the robot slides on the surface in all the locomotion movements and the encoders read the rotation data from the wheels even without displacement. Therefore, when the robot is climbing, the error in locomotion angle increases due to skidding and slippage.

**Figure 19:**
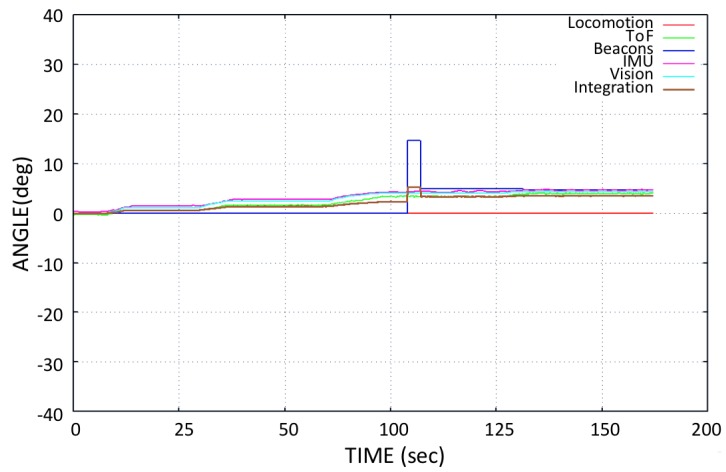
Flat movement test: Moving the *Mantis v2* along the window parallel to the frame in the base, in an open loop.

When making the transition from one window panel to the other, the robot is placed perpendicular to the frame of the window and parallel to the axis of the lower structure. This is true for most window types, which are mostly square. For this application, the robot is currently tele-operated during navigation. However, a system was developed that starts the automatic transition by detecting the metallic frame of the window, e.g., in [36] by using inductive sensor installed at the base of robot. During the transition phase, the blower of the pad is turned off to detach it from the window surface. When moving forward, due to the moment generated by the weight of the lifted pad, the robot turns slightly downwards, as shown in Figure 19, misaligning the robot for a proper transition. For thin frames, it does not affect the transition; however, if the frame is too wide, it can destabilize the robot’s fixation to the window surface and it may cause the *Mantis v2* to fall down. Figure 19 shows the behavior of the robot’s angle during the transition phase. During the transition phase, the robot receives a command to move straight in a flat angular position (zero rotation angle) over the frame. However, the robot slides due to the weight of the lifted pad and, at the same time, it tries to move straight at a zero angular position.

The locomotive encoder sensory system does not detect the wheel rotation because it is not possible to estimate the sliding of the robot on the window using wheel encoders. A significant variation in the beacon sensor orientations is also observed during this test. However, the error is corrected by the sensory integration, as shown in Figure 18 and Figure 19. During navigation test, some sensors do not work properly, as previously mentioned. Given the conditions of the sensory system, it is desired that the data of the sensors that do not work properly should not contemplated for the integration of the orientation of the robot. For this, it is necessary to estimate the orientation error that exists in different sensors. A comparison of the orientation from different sensory systems is made with the IMU sensor (*VN-100*), since it is a highly accurate AHRS sensor [41]. The Root Mean Square Error (RMSE) is calculated by taking IMU *VN-100* orientation angle as a reference, according to Equation (14):

(14) $$RMSE=\sqrt{\frac{1}{n}\sum_{j=1}^{n}{\left(IMU_{j}-sensor_{j}\right)}^{2}}.$$

**Figure 20:**
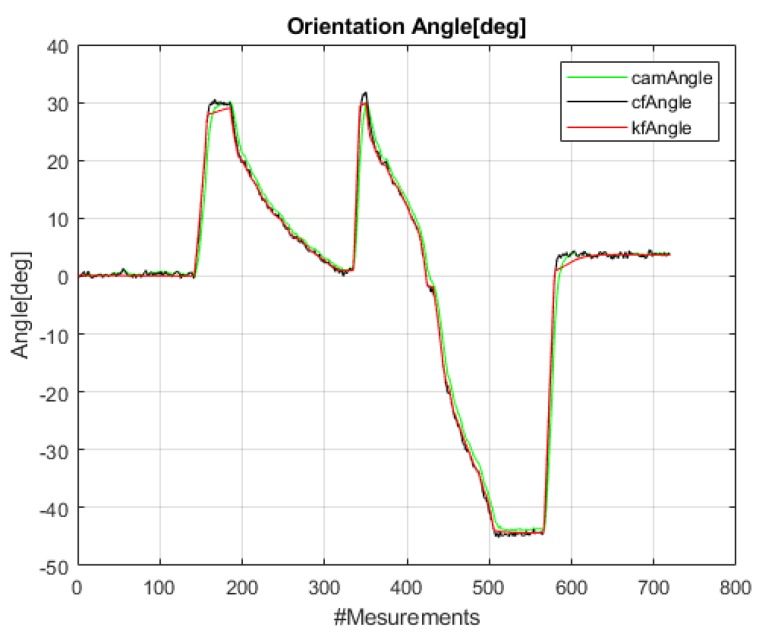
Rapid turning test: Orientation angle of the *Mantis v2* ($\theta_{m}$) estimated using accelerometer and gyro fusion in the complementary filter (CF), Kalman filter (KF), and vision system.

Figure 20 provides a separate test with rapid and high rotations of ≈$-45^{\circ }$ to $30^{\circ }$ of the *Mantis* robot during navigation on the window panel. The sensor integration results utilizing data from the IMU’s raw sensor data, i.e., gyro and accelerometer data fused using CF and KF, are plotted and compared with the reference angle (*VN-100*) in Figure 20. Note that vision-based angle tracking is also plotted in this rapid-turning test to check its stability and accuracy.

**Figure 21:**
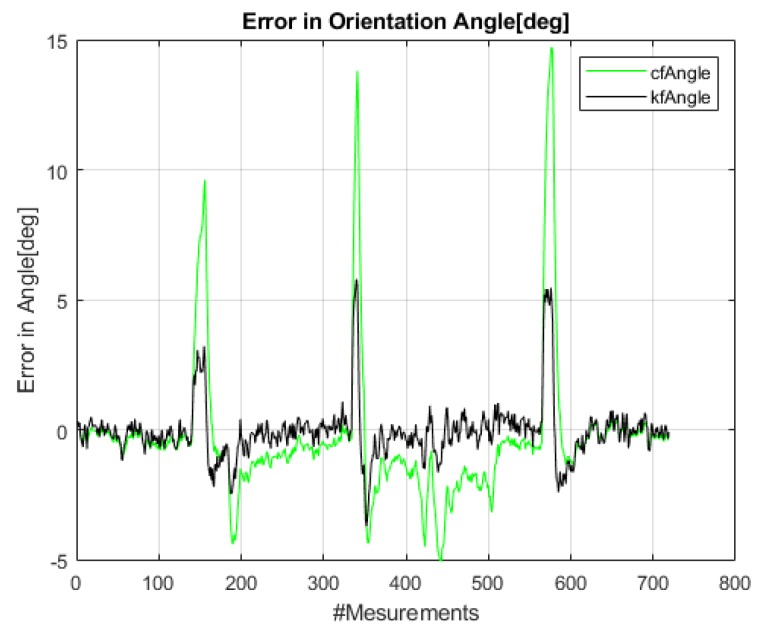
Error in the integrated angle of the *Mantis v2*.

Figure 21 below is a plot of the error in orientation angles. This plot is obtained by differencing the angle values between reference the AHRS (*VN-100*) and the designed algorithms [41], i.e., camera-based angle, CF, and KF. It is observed that the CF has some high jumps when the robot is rapidly turning clockwise or counterclockwise, whereas the KF faithfully tracks the rotation angle of the *Mantis* robot. The maximum error in orientation angle error from the raw IMU sensor’s data remains within $\pm 2^{0}$. The RMSE for the CF is 2.75 degrees and for the KF is 1.14 degrees in this rapid-turning test.

Table 2 shows the RMSE data obtained by comparing the reference *VN-100* IMU orientation data and the sensory systems in the *Mantis v2*. The table shows the experiments taken in static angular positions (–60${}^{\circ }$ to +60${}^{\circ }$) of the robot and in the three main types of dynamic tests, which are: The navigation test of the robot on the window, horizontal locomotion of $0^{\circ }$ (flat move), and the transition between window panels.

In the error data table above, it can be observed that the orientation based on wheel encoder data shows the biggest errors, and, in the static position, it is not possible to get angular data from wheel encoders. The beacon sensors have a delay in the signal update, which results in the orientation of the robot being traversed with respect to the rest of the sensors. The delay in the beacon signal can cause erroneous data with respect to the real orientation of the robot, putting at risk the integrity of the robot, especially during navigation from one panel to another.

As a conclusion of this analysis, the average orientation errors in the static tests of the different sensory systems in this work are given in the Table 3.

**Figure 22:**
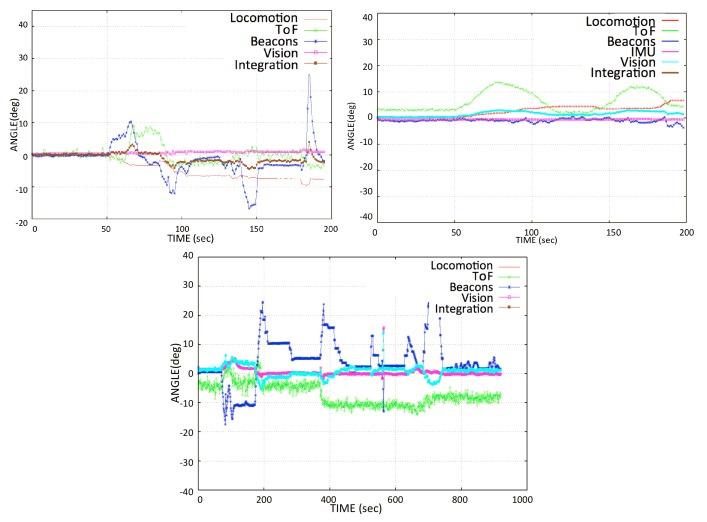
Orientation error with respect to the *VN-100* IMU sensor: Navigation, flat movement, and window panel transition tests.

The orientation angle results of the various sensors used in the *Mantis v2* robot are compared with those of the *VN-100* IMU, a highly accurate AHRS sensor, in this article, and the error plot is given in Figure 22.

## 6. Conclusions

In this article, an evaluation of different orientation sensors is made for a façade-cleaning robot. Each orientation sensor has its own merits and demerits when compared in terms of accuracy, latency, and noise characteristics. Since each of these sensors has various limitations, if only one type of sensor is used, the probability of erroneous orientation angles, unreliability, and system failure will increase. Sensory integration proved a better error correction method for utilizing data coming from the various types of conventionally used sensors. The resilience of the sensing system for orientation is important, as, if the sensor system goes wrong, the risk of unsafe situations is very high.

During experimentation in this work, it was observed that the ToF sensory system works only when the windows have square shapes and have a relatively high (about 4 cm) boundary frame to be detected. Likewise, the sensing range will be limited only to the lower part of the window frame. Wheel encoders showed the worst behavior in orientation estimation in this work, primarily due to the frequent skidding and slippage of the *Mantis v2* robot. With the IMU sensor alone, the cumulative error in orientation was observed to increase with the passage of time; however, sensor fusion with the CF or 1D Kalman filter is a viable solution. For the beacon sensory system, it was observed that a positional error of about ±2 cm occurs; coupled with the update speed, an orientation error is generated between the real angular position of the robot and that calculated by the beacon sensor, with a delay of up to 5 s, which can cause collisions or unnecessary movements in the robot during navigation. Vision-based angle calculation gave good results in this work. However, to install a camera which can track the robot during the entire operation in outdoor conditions is a difficult task.

In dynamic tests, vision and sensor integration consistently shows lower errors. The lowest error (≈$0.8^{\circ }$) in orientation angle is observed in vision sensor, as it has no effect of robot skidding, time elapse, or signal delay. Multi-sensor integration reduces errors in orientation angle by fusing data with algorithms like data averaging. It is observed that, when lifting any side modules of the robot off the glass surface during the transition between two window panels, the weight of the module slightly bends the robot’s structure. This curvature modifies the orientation of the robot without being detected by the sensors.

As part of future work, the locomotive system will be changed to reduce skidding and slippage of the robot. The materials used in the main structures will be replaced entirely by carbon fiber. We are currently developing a mothership system that supplies energy and supplements for the cleaning robot. Moreover, the mothership can be used to install vision systems that can track the robot’s position during operation. Localization and mapping tasks will be carried out using cameras, IMUs, and Lidar fusion. 

## Figures and Tables

**Table 1:** Distance matrix used in the MarvelMind software to determine the position of the mobile beacon (Bm).

ID	ID1	ID2	ID3	ID4	ID5	ID6
ID1	-	2.75	-	-	1.77	3.27
ID2	2.75	-	$D_{3-2}$	$D_{4-2}$	3.27	1.77
ID3	$D_{1-3}$	$D_{2-3}$	-	$D_{4-3}$	$D_{5-3}$	$D_{6-3}$
ID4	$D_{1-4}$	$D_{2-4}$	$D_{3-4}$	-	$D_{5-4}$	$D_{6-4}$
ID5	1.77	3.27	$D_{3-5}$	$D_{4-5}$	-	2.75
ID6	3.27	1.77	$D_{3-6}$	$D_{4-6}$	2.75	-

**Table 2:** Root mean square error (RMSE) comparison of orientation data (degrees).

Ref. Angle	Odometry	ToF	Beacons	Vision	Integration
0	NA	0.799	0.196	0.31	0.137
10	NA	0.391	0.501	0.772	0.333
20	NA	1.25	0.814	0.73	0.559
30	NA	2.071	1.011	0.025	0.217
40	NA	NA	0.971	0.854	0.023
45	NA	NA	2.772	0.145	0.583
60	NA	NA	0.076	0.061	0.003
−10	NA	0.162	0.731	0.4	0.098
−20	NA	0.387	1.763	1.662	0.607
−30	NA	0.884	1.669	1.98	0.239
−40	NA	1.731	0.527	0.082	0.468
−45	NA	0.427	0.102	0.373	0.031
−60	NA	NA	0.843	0.132	0.142
Nav. (30 to −20)	2.093	0.124	1.533	0.722	0.956
Flat Move	2.634	5.588	0.944	0.543	1.347
Transition	17.506	3.035	0.091	0.382	0.203

**Table 3:** Details of the orientation sensors and achieved results (degrees).

Orientation System	Properties	Achieved Results (deg.)
Encoder	Encoder 64 pulse resolution	10.562
ToF	range = 2 m, resolution 1 mm, accuracy = 3%	0.90
Sonar Beacon Sys.	High precision (2 cm),range = 50 m, location update @ 25 Hz	0.92
Camera	1920 × 1080 pixels @ 30 fps, 78 deg FoV	0.58
IMU	0.5/1.0 deg. Static/Dynamic Pitch and Roll @ 400 Hz	0.26

## Abbreviations

The following abbreviations are used in this manuscript:

ROS	Robot Operating System
IMU	Inertial measurement unit
ToF	Time-of-Flight
KF	Kalman filter
CF	Complementary filter
AHRS	Attitude and heading reference system
PLA	Polylactic acid
TPU	Thermoplastic polyurethane-elastomere
Bm	Mobile beacons
Bs	Stationary beacons

## Appendix A

### Appendix A.1. Complementary Filter

The complementary filter (CF) is a simple way to fuse data from two sensors, e.g., accelerometer and gyros in this work. In the short term, the gyroscope is used to get the angular position because it is very precise and not susceptible to external forces, such as gravity force, whereas, in the long term, accelerometer data is used because it does not drift with time. The simplest form of CF is given below in Equations (A1)–(A3):

(A1) angle=α∗GyroAngle+(1-α)∗accelAngle,

where

(A2) $$accelAngle=\arctan\left(\frac{a_{y}}{-a_{z}}\right),$$

and

(A3) $$GyroAngle=GyroAngle+g_{x}*dt,$$

where dt is the sampling time and alpha (α) is a tuning parameter, with values ranging from 0–1.

In every iteration, the orientation angle is updated with the new gyroscope measurement. To eliminate the effects of external forces on accelerometer measurements, the magnitudes of the accelerometer readings are checked in each iteration. If the magnitude of the accelerometer measurements is less than a certain threshold (as in Equation (A4)),

(A4) $$\sqrt{\left(a_{x}^{2}+a_{y}^{2}+a_{z}^{2}\right)}\leq Threshold,$$

then the orientation angle is updated with the accelerometer data by adding (1-α) times the angle calculated with the accelerometer measurement. This will ensure that the measurement will not drift with time, and will be very accurate in the short term. The CF filter is easy to understand and easy to implement, making it suitable for low-cost embedded systems on lightweight platforms like the *Mantis v2*.

### Appendix A.2. One-Dimensional Kalman Filter

As for the façade-cleaning robot, the only orientation angle we have to track is the heading angle of the robot on the vertical glass wall. Only one-dimensional angle estimation is required to control the motion of the robot in a 2D environment.

The one-dimensional Kalman filter (KF) used in this work is briefly explained here. The KF model assumes that the state of a system at the time (t) evolved from the prior state at the time (t-1) according to the general equations of the KF:

(A5) x(t)=F∗x(t-1)+B∗u(t)+W,

where **x**(t) (Equation (A5)) is the state vector containing the terms of interest for the system (e.g., orientation of *Mantis v2* and bias term of the gyro’s x-axis) at time *t*, **u**(t) is the vector containing the control inputs (if any), **F** is the state transition matrix which applies the effect of each system state parameter at time (t-1) on the system state at the next time step (t), **B** is the control input matrix which applies the effect of each control input parameter in the vector **u**(t) on the state vector, and **W** is the vector containing the process noise terms for each parameter in the state vector. The process noise is assumed to have a zero-mean Gaussian distribution with co-variance matrix **Q**.

The measurement model used in the linear KF, in general, is given as follows in Equation (A6):

(A6) z(t)=H∗x(t)+v,

where **z**(t) is the vector of measurements, **H** is the transformation matrix that maps the state vector into the measurement domain, and **v** is the vector containing the measurement noise for each observation in the measurement vector. Like the process noise, the measurement noise is assumed to be zero-mean white Gaussian noise with co-variance matrix **R**.
